#  Emerging Infections Program Efforts to Address Health Equity

**DOI:** 10.3201/eid2109.150275

**Published:** 2015-09

**Authors:** James L. Hadler, Duc J. Vugia, Nancy M. Bennett, Matthew R. Moore

**Affiliations:** Connecticut Emerging Infections Program, Yale School of Public Health, New Haven, Connecticut, USA (J.L. Hadler);; California Emerging Infections Program, California Department of Public Health, Richmond, California, USA (D.J. Vugia);; New York Emerging Infections Program, University of Rochester Medical Center, Rochester, New York, USA (N.M. Bennett);; Centers for Disease Control and Prevention, Atlanta, Georgia, USA (M.R. Moore)

**Keywords:** race, ethnicity, health status disparity, socioeconomic factors, poverty, social determinants of health, inequalities, Emerging Infections Program, EIP

## Abstract

The Emerging Infections Program (EIP), a collaboration between (currently) 10 state health departments, their academic center partners, and the Centers for Disease Control and Prevention, was established in 1995. The EIP performs active, population-based surveillance for important infectious diseases, addresses new problems as they arise, emphasizes projects that lead to prevention, and develops and evaluates public health practices. The EIP has increasingly addressed the health equity challenges posed by Healthy People 2020. These challenges include objectives to increase the proportion of Healthy People–specified conditions for which national data are available by race/ethnicity and socioeconomic status as a step toward first recognizing and subsequently eliminating health inequities. EIP has made substantial progress in moving from an initial focus on monitoring social determinants exclusively through collecting and analyzing data by race/ethnicity to identifying and piloting ways to conduct population-based surveillance by using area-based socioeconomic status measures.

## Abstract 2

The EIP has expanded efforts to measure health inequities by using area-based socioeconomic analyses.

Describing health disparities and achieving health equity have been priorities of the national public health agenda for the past 20 years. One of the 2 goals of Healthy People (HP) 2010, the public health agenda for 2000–2010, was to “eliminate health disparities among different segments of the population, including differences that occur by gender, race or ethnicity, education or income, disability, geographic location, or sexual orientation” (*1*). In addition, HP 2010 included a related public health infrastructure objective (23.4), to track HP 2010 objectives by each population group. HP 2020, the agenda for 2010–2020, specifically added mention of social determinants of health. It reframed the goal and the related infrastructure objective, the former as “Achieve health equity, eliminate disparities, and improve the health of all groups,” and the latter, now Public Health Infrastructure Objective (7.1), as “Increase the proportion of population-based Healthy People 2020 objectives for which national data are available for all population groups” with a specific subobjective (7.3) “by socioeconomic status” (*2*).The World Health Organization, in a similar vein, recently recognized that addressing the social determinants of health was a key priority to eventually achieving health equity (*3*).

The Emerging Infections Program (EIP), established by the Centers for Disease Control and Prevention (CDC) in 1995, is a network that now includes 10 state health departments and their collaborators in local health departments, academic institutions, other federal agencies, and public health and clinical laboratories, with a catchment area of ≈44 million persons (*4**–**7*). In addition to performing active, population-based surveillance for important infectious diseases, EIP activities are intended to be flexible and address new problems as they arise, answer critical public health questions, emphasize projects that lead to prevention, and develop and evaluate public health practices. In this context, the EIP has increasingly taken on the challenges posed by HP 2010 and HP 2020, moving from a focus on monitoring social determinants exclusively through collecting and analyzing data by race/ethnicity to identifying and piloting ways to conduct population-based surveillance by using socioeconomic status (SES) measures with an ultimate focus on working toward health equity.

Most data collected by EIP sites comes from laboratory-based surveillance for bacterial, parasitic, and viral diseases, which does not include individual-level SES information. Missing data, especially ethnicity, is a consistent challenge. However, because residency in an EIP catchment area is a requirement for inclusion in surveillance, and to enable deduplication of multiple reports, addresses of residence for individual case-patients are collected at the time of diagnosis, making it possible to link cases to specific census tracts. With linkage to census tract, a wealth of data on census tract–level SES status indicators (e.g., poverty, education level, crowding) becomes available. Seminal work done by Nancy Krieger and colleagues in the Public Health Disparities Geocoding Project found that these census tract–level SES measures, especially low SES status (usually the lowest quartile or quintile of each measure), often predict disease incidence and mortality rates (*8**,**9*) and do so within and across groups defined by race/ethnicity. These authors recommended routine use of area-based SES measures in disease surveillance to describe and monitor, over time, disparities by SES, particularly poverty as measured by the percentage of persons in a census tract who lived below the federal poverty level.

In this article, we describe the evolution of EIP involvement in monitoring health disparities and in working toward health equity. These efforts began with a focus on race/ethnicity and, more recently, have included the piloting use of area-based SES measures under the guidance of a Health Equity Working Group.

## EIP and Health Disparities

To date, EIP population-based surveillance data (from single or multiple sites) have been used to describe racial and socioeconomic disparities for invasive pneumococcal disease (IPD), invasive group B *Streptococcus* (GBS) disease, invasive group A *Streptococcus* (GAS) disease, influenza-associated hospitalizations, and several other diseases (*10**–**32*).

### IPD (*Streptococcus pneumoniae*)

Racial disparities in the incidence of IPD have long been described (*10*). Before the 7-valent pneumococcal conjugate vaccine (PCV7) was introduced in the United States in 2000, IPD rates among blacks had been documented by the EIP to be approximately twice that of whites, and this disparity was seen in all age groups (*11*). After PCV7 was introduced, the racial disparity in IPD, caused by bacterial serotypes included in PCV7, was eliminated for children <5 years of age, and rates among white children decreased below the HP 2010 target of 46 cases per 100,000 population by 2001 (*12**–**14*). Rates for black children met this goal a year later. However, incidence rates for those >5 years of age remained higher among blacks.

The most recent analysis of national EIP IPD data tracked the effects of trends in PCV7-type and non–PCV7-type IPD rates on racial disparities (*15*). Although incidence of IPD caused by PCV7-types was nearly eliminated in blacks and whites after PCV7 was introduced, rates of non–PCV7-type IPD increased in both races so that, by 2009, non–PCV7-type IPD incidence among blacks was still much higher than that among whites, in all age groups (*15*). Research has suggested that these findings may be due to a higher prevalence of underlying conditions among blacks (e.g., asthma, diabetes, HIV/AIDS) and lower SES (*16**–**18*).

The first analysis of IPD data that controlled for SES by using census tract measures was a study of 1994–1997 rates in San Francisco County, published by the California EIP (*19*). In a Poisson model, black race and living in census tracts with low median household income were both highly significantly associated with higher IPD rates. In an analysis of 2003–2004 bacteremic pneumonia (caused by *S. pneumoniae*, *Haemophilus influenzae*, GAS, and GBS), data from 9 EIP sites showed that the rates were highest among US adults living in the poorest census tracts (with >20% of persons living below federal poverty level) and among blacks (*20*). In models that controlled for age, census tract poverty level, and EIP site, “racial disparities in incidence were reduced but remained significant.” This article was notable for using geocoded EIP surveillance data and linking such data to the 2000 US census tract data for the analyses, following the method of the Public Health Disparities Geocoding Project (*8*).

The Connecticut EIP also used this method to analyze and describe the changing disparities in IPD incidence rates in Connecticut during 1998–2008 by SES and by race/ethnicity (*21*). The authors found that before the introduction of PCV7, persons living in high-poverty census tracts had a much higher incidence of IPD than their counterparts in low-poverty census tracts. After PCV7 was introduced, these differences nearly disappeared for those infected with PCV7 serotypes but increased for those infected with non-PCV7 serotypes.

### Invasive GBS Disease

Racial disparities have also been described for invasive GBS disease. In the late 1980s, black infants in metropolitan Atlanta were found to have a higher risk for early- and late-onset GBS disease than white infants (*22*), and black adults had higher rates of invasive GBS infections than white adults (*23*).

Coincident with active efforts in the mid-1990s to provide antimicrobial prophylaxis to pregnant women at risk of transmitting GBS to their newborns, EIP data documented that early-onset invasive GBS disease had decreased substantially by 1998 for both white and black neonates, but the incidence in black neonates remained higher than that in whites (*24*). The 2010 national health objective of 0.5 cases per 1,000 live births has been reached among white neonates since 1998; although the incidence for black neonates has been approaching this goal, it had not been achieved by 2003 (*25*). Indeed, through 2006, EIP surveillance data showed that the rate of early-onset invasive GBS disease had increased for black infants, particularly preterm black infants, widening the gap between black and white infants (*26**,**27*). During 1990–2005, the incidence of late-onset neonatal GBS disease, which intrapartum antibiotic prophylaxis does not prevent, was also higher among black infants than among white infants (*28*).

In addition to disparities for GBS disease, disparities are found for other causes of neonatal sepsis. During 2005–2008, EIP surveillance documented that rates of early-onset neonatal sepsis (caused by GBS, *Escherichia coli*, viridans streptococci, and other bacteria) among black preterm infants were >10 times those of nonblack term infants (5.14 vs. 0.4 cases/1,000 live births) (*29*). The authors of this study commented that race is likely a surrogate for social determinants of health that contribute more broadly to disease disparities.

### Invasive GAS Disease

The epidemiology of invasive GAS disease in the United States was described by using 1995–1999 population-based surveillance data from several EIP sites (*30*). The incidence of invasive GAS disease among blacks was 1.6 times higher than incidence among other racial groups. The higher incidence of invasive GAS disease among blacks was confirmed in another analysis of 1989–1999 surveillance data from the California EIP (*31*). The authors suggested that race may be a surrogate marker for other, unmeasured, factors such as access to health care.

### Influenza

The Connecticut EIP analyzed influenza-associated hospitalizations and census tract SES following the process used by the Public Health Disparities Geocoding Project (*8*). In one analysis, 2003–2010 influenza-associated hospitalization cases among children <18 years of age were geocoded and linked to 2000 census tract SES data (*32*). The mean annual incidence of influenza–associated hospitalizations for children in high-poverty and high-crowding census tracts was at least 3 times greater than that in low-poverty and low-crowding tracts. This disparity could not be fully explained by prevalence of underlying conditions or receipt of influenza vaccination. Incidences of influenza-associated hospitalization among black and Latino children were 3.4 and 3.0 times higher, respectively, than among white children.

In another analysis, 2007–2011 influenza-related hospitalization cases of adults >18 years old were geocoded and linked to 2006–2010 American Community Survey data for census tract SES measures (*33*). Again, a statistically significant trend was found: the incidence of influenza-related hospitalizations increased as SES decreased (or poverty increased), for each influenza season and within each racial/ethnic group. Black and Latino adults had higher influenza hospitalization rates than white adults within each SES group, and rates for women were higher than those for men for each age group. The study authors noted that systematic efforts are needed to achieve higher influenza vaccination rates in low SES neighborhoods and among women.

### Campylobacteriosis

The Connecticut EIP geocoded 1999–2009 cases of campylobacteriosis and linked them to 2000 census tract data to analyze for case-patient SES (*34*). For children <10 years of age, campylobacteriosis rates increased as census tract poverty level increased, but for children >10 years of age and for adults, rates decreased as census tract poverty level increased. The authors stated that children living in poorer census tracts could conceivably have a higher rate of exposure to *Campylobacter* spp. in the home, although this possibility needed to be verified.

### Cervical Cancer Precursors

Again using methods of the Public Health Disparities Geocoding Project, the Connecticut EIP linked 2008–2009 Connecticut cases of cervical intraepithelial neoplasia grade 2 or higher and adenocarcinoma in situ (CIN2+/AIS), cervical cancer precursors, with poverty level as determined from 2000 census tract data (*35*). The authors found that, overall, higher rates of CIN2+/AIS were associated with higher levels of poverty and that the association of higher proportions of African American residents with poverty was the strongest and most consistently associated measure. Among women 20–24 years of age, however, CIN2+/AIS rates were inversely associated with poverty, a finding suggesting that screening rates are higher among those living in SES census tracts where income level is higher.

EIP population-based surveillance data analyses have contributed to identification or confirmation of race- and SES-based health disparities for several diseases under EIP surveillance. Linking geocoded surveillance data to census tract SES measures has shed further light on the influence of neighborhood poverty on some of these diseases. Notably, when interventions have decreased infection rates of IPD and invasive GBS significantly, some racial disparities remained. Although the underlying reasons for racial disparities associated with these diseases await further investigation, identifying such disparities on the basis of SES provides opportunities for focusing prevention efforts to populations defined by SES rather than solely by race/ethnicity.

## EIP Health Equity Working Group

In 2012, the EIP commissioned an external review to seek advice about future directions and strategies. Among other issues, the external review panel was asked specifically to identify areas for increased emphasis considering the extant portfolio of work, the strengths and composition of the network, and key public health issues involving infectious diseases. The panel responded: “EIP should develop and implement a plan for studying the role and [causal] pathways of underlying determinants of health in creating infectious disease disparities, and the extent to which these pathways are similar or different across diseases” (EIP External Review Report, 2012). This recommendation was discussed extensively at the 2013 meeting of the EIP Steering Committee and, as a result, the EIP Health Equity Working Group was formed. Members of the Working Group include interested collaborators from the EIP sites as well as CDC staff. The goals of the working group are to describe disparities and the contributions of various social determinants of health, to target and evaluate the effects of interventions aimed at reducing disparities, and, to the extent possible, to develop and test hypotheses regarding the causal pathways that lead to disparities in conditions studied by the EIP.

To focus on the social determinants of health, the working group determined that it was critical to move beyond an analysis exclusively based on race/ethnicity and to take advantage of having the residence address of case-patients. The addresses can be geocoded routinely and linked to census tract-level socioeconomic data as described (*8*). An approach using area-based SES data to describe health disparities provides certain advantages over EIP’s previous approach based on race/ethnicity (*12*). Although race/ethnicity and SES are associated in the United States, use of race/ethnicity as a proxy for SES has distinct limitations. Race and especially ethnicity are often missing from inpatient medical records, requiring the use of complex methods to impute unknown values (*8*). In addition, unlike income or educational level, race/ethnicity is not modifiable, and it has been biologically causally linked to disparities only for certain conditions (e.g., sickle cell disease) (*36*). Further, SES is a determinant of health within groups defined by race/ethnicity, something not measured when race/ethnic group is the variable of analysis (*8*). Finally, using race/ethnicity as a proxy for SES in the absence of other measures fails to capture the full spectrum of social determinants of disease and makes it difficult to accurately target interventions.

One of the strengths of the EIP is that methods are standardized among the 10 geographically distributed sites. The first order of business for the Health Equity Working Group was to develop a standardized protocol for geocoding cases to the census tract level. Case-patients that are college students or residents of long-term care facilities, for example, often have at least 2 addresses to which they could be geocoded, and this protocol ensures they are geocoded in the same way by all EIP sites. A variety of geocoding software is now available, and it varies in its ability to geocode cases to the rooftop level, a method which is necessary to accurately assign addresses to census tracts (*37*). Although individual EIP sites are not required to use the same software, the protocol does require the use of software capable of geocoding to the rooftop level. Privacy concerns have been addressed by limiting retention of address information to the local EIP sites. For activities that might involve small numbers of potentially identifiable case-patients, additional methods are available to protect confidentiality (*38*). In addition, the EIP affords investigators an opportunity to use the best possible analytic approaches to better define the influence of a variety of social determinants.

## Future Directions

**Figure 1:**
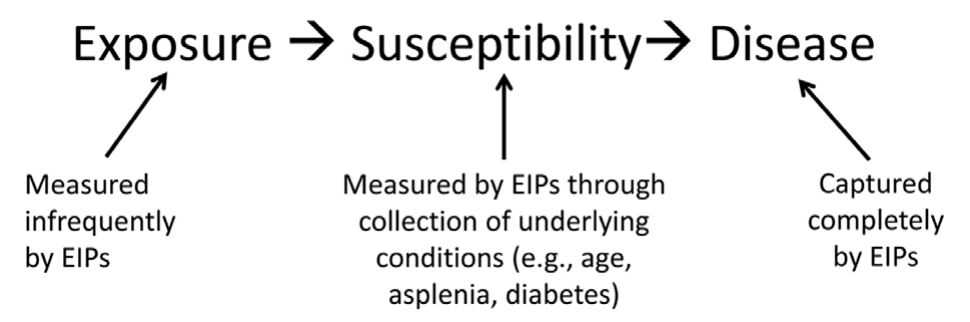
Simplified causal pathway previously accessible by using Emerging Infections Program (EIP) data.

Although the EIP has substantial experience in evaluating the effects of public health interventions, including some that have reduced racial disparities, the EIP has less experience developing and testing hypotheses regarding the causal pathways that lead to health disparities in the first place. Instead, the EIP has historically collected basic demographic data (age, sex, race/ethnicity) and clinical data available from the inpatient medical record (date of hospitalization and presence of underlying conditions such as diabetes and heart disease). These limited data lend themselves to only a simple causal pathway (Figure 1). The causal pathway to direct exposure to infections monitored by the EIPs, such as exposure to respiratory pathogens, is extremely difficult to determine in nonoutbreak settings. On the other hand, assessing individual-level susceptibility, such as that conferred by certain underlying conditions, is relatively straightforward to measure through review of medical records. But this simplistic model misses separate but important dimensions of risk of disease.

**Figure 2:**
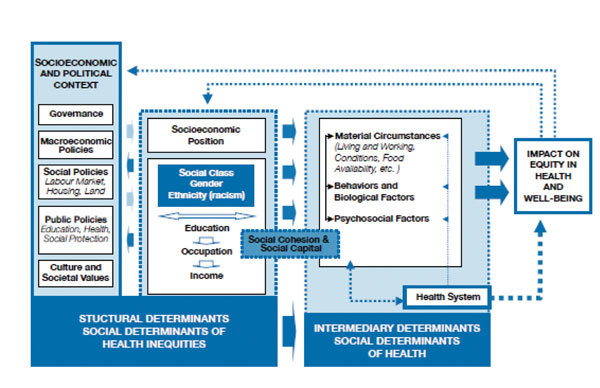
Framework for considering social disparities of health determined by the Commission on Social Determinants of Health, World Health Organization (*3*).

In contrast, the World Health Organization’s Commission on Social Determinants of Health has adopted a more complex framework for considering the nature of health disparities (Figure 2) (*3*). Under this framework, interventions are aimed at the circumstances of daily life and the structural drivers of disparities. The former includes differential exposure to disease, such as those that occur early in life, those that affect social and physical environments, and work, all of which are associated with differences in social strata. The structural drivers of disparities include the nature and degree of social stratification; biases, norms, and values within societies; global and national economic and social policy; and processes of governance at global, national, and local levels.

The commission also made several recommendations, including one to “measure the problem, evaluate action, expand the knowledge base, develop a workforce that is trained in the social determinants of health, and raise public awareness about the social determinants of health.” To respond to that recommendation, the EIP will need to broaden its scope of expertise and collaborate with new partners. These partners could include, for example, academic investigators who are advancing our understanding of the biologic basis for the effects of social position on risk of disease (*39*) and professional organizations that support new ways of thinking about health disparities (*40*). Such collaborations could shed light on ways in which the EIP could design new studies aimed at understanding the causal pathways leading to health disparities, and they could assist the EIP in becoming a vocal advocate for health equity.
